# Whole-genome sequencing and comparative genomic analysis of *Agrobacterium fabrum* L-11 causing crown gall disease in blueberry

**DOI:** 10.3389/fmicb.2026.1776711

**Published:** 2026-05-06

**Authors:** Han Meng, Yue Qiu, Jinhao Zhang, Haowen He, Juxiang Wang, Dalal Hussien M. Alkhalifah, Lanfang Wei, Waqar Ahmed, Guanghai Ji

**Affiliations:** 1 State Key Laboratory for Conservation and Utilization of Bio-Resources in Yunnan/Key Laboratory of Agro-Biodiversity and Pest Management of Ministry of Education, Yunnan Agricultural University, Kunming, Yunnan, China; 2 College of Agriculture, Anshun University, Anshun, Guizhou, China; 3 Department of Biology, College of Science, Princess Nourah Bint Abdulrahman University, Riyadh, Saudi Arabia; 4 School of Breeding and Multiplication (Sanya Institute of Breeding and Multiplication), Hainan University, Sanya, China

**Keywords:** *Agrobacterium fabrum*, comparative genome analysis, crown gall disease, functional annotation, whole-genome sequencing

## Abstract

**Background and aims:**

Crown gall caused by *Agrobacterium fabrum* is a significant bacterial disease affecting blueberry production worldwide. However, the lack of whole-genome information for this pathogen impedes genetic and molecular analysis of its pathogenic mechanisms.

**Method and results:**

To address this knowledge gap, *A. fabrum* L-11 was isolated from blueberry-growing areas in Qujing City, Yunnan Province, and its genome was sequenced using the Oxford Nanopore platform. Additionally, a comparative genome analysis was conducted with strains of *A. radiobacter* K84, *A. fabrum* 1D132, and *A. fabrum* C58 to understand its pathogenic mechanism, evolutionary path, and virulence factors. The complete genome of *A. fabrum* L-11 is 5.70 Mb with a GC content of 59.0%, consisting of a 2.93 Mb linear chromosome, a 2.02 Mb circular chromosome, and four large plasmids ranging from 0.12 to 0.232 Mb (total plasmid content 0.743 Mb), an exceptional feature among *Agrobacterium* strains. The genome includes 5,385 genes, comprising 5,245 protein-coding sequences (CDS) and 140 RNA genes (54 tRNAs, 12 rRNAs, and 74 other non-coding RNAs). Functional annotation revealed significant enrichment in multiple metabolic pathways such as ABC transporters, amino acid biosynthesis, and carbon metabolism. The genome encodes a broad range of carbohydrate-active enzymes (CAZymes), including glycoside hydrolases and glycosyl transferases, indicating advanced capabilities in carbohydrate metabolism. A comparative genome study showed that *A. fabrum* L-11 differs from other strains in terms of virulence factors, GC content, chromosomal number, and plasmid count. Virulence factor analysis identified 1,071 potential virulence-related genes, including those associated with secretion systems, ABC transporters, and lipopolysaccharide production. With notable differences in opine usage compared to other strains, the Ti plasmid analysis revealed essential genes involved in virulence and tumor formation.

**Conclusion:**

*Agrobacterium fabrum* L-11’s comprehensive genome analysis offers valuable new insights into its metabolic abilities, pathogenicity mechanisms, and evolutionary links with other Agrobacterium species. This genetic information will serve as a foundation for research on host-pathogen interactions and assist in developing biocontrol strategies for crown gall disease in blueberries, leading to more effective disease management.

## 1 Introduction

Crown gall disease, caused by bacteria of the genus *Agrobacterium*, is one of the most economically damaging bacterial diseases affecting perennial crops worldwide (Otten et al., 2008). Among the various *Agrobacterium* species, *Agrobacterium fabrum* has become a particularly important pathogen of fruit crops, including blueberries, where it causes distinctive tumorous galls that hinder plant growth and yield (Pulawska, 2010). It has been reported that *Agrobacterium* can infect over 1,000 different plant species across most families of dicotyledons (Shiboleth and Tzfira, 2012). The disease was first identified more than a century ago, yet it still threatens global agriculture, with recent reports showing its spread into new regions and host species (Kuzmanović and Puławska, 2019). This expansion of host range is illustrated by recent discoveries of new crown gall pathogens. For example, Kuzmanović et al. (2018b) identified *Rhizobium tumorigenes* as a cause of crown gall on rhododendron, and later studies showed this pathogen can also infect blueberries (Kuzmanović et al., 2019). A recent study reported that *A. fabacearum* also causes crown gall disease in kiwifruit plants in Guiyang, Guizhou Province, China (He et al., 2021). This highlights the evolving nature of *Agrobacterium*-related diseases and the potential for new pathogens to expand the host range of crown gall disease.

Blueberry (*Vaccinium* spp.) has gained worldwide recognition as an important fruit crop due to its high nutritional content and health benefits, including antioxidants, anti-inflammatory effects, and neuroprotection (Singh et al., 2025). Over the past decade, global blueberry production has increased significantly, with key regions including North America, Europe, Chile, and China (Lobos and Hancock, 2015). However, this expansion has resulted in increased disease pressure, particularly from soil-borne pathogens like *Agrobacterium* (Fulcher et al., 2015). Crown gall disease in blueberries was first identified in the 1990s and has since been reported across major blueberry-growing regions worldwide. It causes significant economic losses by reducing plant vigor, decreasing yields, and increasing mortality in nurseries and production fields (Burr et al., 1998). *A. fabrum* primarily infects the host through wounds and occasionally infects the ground rhizome or the stem connection. In the early stages of infection, blueberry root galls are round and yellow-white. Later, the galls become more prominent and change from yellow-brown to dark brown (Kuzmanović et al., 2018a). The galls are lignified, rough, nearly round or irregular in shape, with diameters of 2–3 cm and up to 10–15 cm in severe cases (Wang et al., 2000). After infection with the crown gall pathogen, water and nutrient absorption in the plant roots is blocked, and root development is delayed (Cubero et al., 1999). The plant grows slowly in the late stage of the disease, and the leaves turn yellow, wither, or even die (Kuzmanović et al., 2019). The occurrence of crown gall disease is closely related to temperature and humidity, soil physical and chemical properties, grafting methods, underground pests, and other factors (Li et al., 2015).

The virulence of *Agrobacterium* species is influenced by a complex set of factors that have been extensively studied for over 40 years. The key feature of *Agrobacterium* pathogenesis is its ability to transfer and insert a piece of plasmid DNA (T-DNA) from the bacterial tumor-inducing (Ti) plasmid into the plant’s genome (Hooykaas, 2023). This process illustrates a rare case of inter-kingdom DNA transfer and has served as a fundamental basis for plant genetic engineering (Faure, 2021). The T-DNA region contains genes that disrupt plant hormone production and processing. Auxin synthesis involves the enzymes *iaaM* (tryptophan monooxygenase) and *iaaH* (indole-3-acetamide hydrolase), which work together to convert tryptophan into indole-3-acetic acid (IAA) (Zhang et al., 2019). Overproduction of cytokinin occurs because of *ipt* (isopentenyl transferase), the enzyme responsible for the rate-limiting step in cytokinin biosynthesis. The interaction of auxin and cytokinin promotes uncontrolled cell division and tumor development (Sakakibara et al., 2005). The T-DNA also encodes opine synthases, such as *ags* (agropine synthase), *nos* (nopaline synthase), and *ocs* (octopine synthase). These enzymes facilitate the production of opines, specialized amino acid or sugar derivatives that serve as nutrient sources exclusively for the bacteria involved (Vladimirov et al., 2015).

Genes on the bacterial chromosome are vital for successful infection. The genes *chvA*, *chvB*, and *chvE* help bacteria attach to plant cells (Tzfira and Citovsky, 2000). *ChvA* and *ChvB* are necessary for making and exporting cyclic β-1,2-glucans, which are important for osmotic adaptation and attaching to plant cell surfaces. ChvE is a sugar-binding protein that enhances *vir* gene induction by interacting with VirA when plant sugars are present (Lepek et al., 1990). The *att* genes regulate polar attachment to plant cell surfaces (Matthysse, 2014), while the *cel* genes enable cellulose production, which is essential for biofilm formation and increased surface colonization (Robledo et al., 2012). Exopolysaccharide (*exo*) genes support surface colonization and offer protection against plant defense mechanisms (Heredia-Ponce et al., 2021). Opines produced in tumor tissues are specifically degraded by the causing Agrobacterium strain, providing it with a nutritional source (Meyer et al., 2019). Opine catabolism genes (*noc*, *occ*, *agc*, *moc*) are located on the Ti plasmid and are usually co-regulated with conjugation genes (*tra*, *trb*). This arrangement links the use of opines to plasmid transfer: opines trigger conjugation, helping spread the Ti plasmids within the tumor environment (Zatyka and Thomas, 1998). In addition to the typical T4SS, many *Agrobacterium* strains also possess Type III (T3SS) and Type VI (T6SS) secretion systems (Czolkoss et al., 2021). T3SS, similar to those found in plant pathogenic bacteria like *Pseudomonas syringae* and *Xanthomonas campestris*, may inject effector proteins that suppress plant immune responses (Alfano and Collmer, 2004). T6SS plays a role in interbacterial competition and might also manipulate eukaryotic hosts, helping in niche establishment and increasing competitive advantage (Gonzalez Moreno and Nishiguchi, 2025).

The availability of full-genome sequences has transformed our understanding of *Agrobacterium* biology and evolution. The first complete genome of an *Agrobacterium* strain, *A. fabrum* C58, isolated from cherry, was published in 2001 (Goodner et al., 2001). Later sequencing of other strains has revealed both conserved traits and considerable diversity within the genus (Tasnawijitwong et al., 2026). All pathogenic *Agrobacterium* strains possess multipartite genomes, which include one or two chromosomes and several plasmids. The *A. fabrum*/*A. tumefaciens* lineage usually has two chromosomes, one circular and one linear (Ramírez-Bahena et al., 2014), while *A. radiobacter* K84 has only a single circular chromosome (Velázquez et al., 2010). *A. vitis* strains generally have two chromosomes and 2–3 plasmids, including Ti plasmids (Palacio-Bielsa et al., 2009). This multipartite arrangement is believed to enhance genome flexibility and promote adaptive evolution by distributing essential and accessory genes across multiple replicon*s* (Palacio-Bielsa et al., 2009; Ramírez-Bahena et al., 2014). Comparative analyses have revealed substantial genomic plasticity and horizontal gene transfer in the evolution of *Agrobacterium*. Genome diversification is driven by genomic islands that contain virulence-related genes, along with mobile genetic elements such as insertion sequences, transposons, integrases, and prophage regions (Qiu et al., 2023).

Although extensive genomic resources are available for *Agrobacterium* strains, no *A. fabrum* strain from blueberries has yet been sequenced and characterized. Therefore, to address this knowledge gap, we conducted whole-genome sequencing and a comparative genome analysis of *A. fabrum* L-11 isolated from blueberries. The main objectives of this study were as follows: first, to assemble and completely sequence the genome of *A. fabrum* L-11, providing a high-quality reference genome for blueberry isolates. Second, to perform comprehensive genomic annotation to identify genes involved in pathogenicity, metabolism, and adaptation, with particular emphasis on virulence determinants. Third, to conduct comparative genomic analysis with previously sequenced strains (*A. radiobacter* K84, *A. fabrum* C58, and *A. fabrum* 1D132) to identify strain-specific features and understand their evolutionary relationships. Fourth, to establish a fundamental genomic resource that enables molecular diagnostics and targeted disease management strategies.

## 2 Materials and methods

### 2.1 Bacterial strain and culture medium conditions

*Agrobacterium fabrum* L-11 was previously isolated from blueberry tissue infected with crown gall disease and stored at our laboratory in the “State Key Laboratory for Conservation and Utilization of Bio-Resources in Yunnan,” Kunming, Yunnan Province, China. The bacterium was cultured on Yeast Extract Mannitol (YEM) agar plates (mannitol 10 g, yeast extract 3 g, beef extract 1 g, dipotassium hydrogen phosphate 0.5 g, sodium chloride 0.2 g, magnesium sulfate 0.2 g, agar 20 g, distilled water 1 L, pH 7.0) (Andharia et al., 2023). The pure culture of *A. fabrum* L-11 was preserved in a 50% v/v glycerol solution at −80 °C for future use.

### 2.2 DNA extraction and genome sequencing

*Agrobacterium fabrum* L-11 was cultured on YEM solid medium by incubation at 28 °C for 36 h. A single colony was then selected from a pure culture, transferred into YEM liquid medium, and incubated at 28 °C for 24 h. Total genomic DNA was extracted using a bacterial DNA extraction kit (Tiangen DP302-02) following the manufacturer’s instructions (Ahmed et al., 2022). After verifying the DNA purity, sequencing analysis was performed by Shanghai Shenggong Bioengineering Co., Ltd. (Shanghai, China) on an Oxford Nanopore sequencing platform. The experimental process followed the standard protocol provided by Oxford Nanopore Technologies (ONT), including sample quality assessment, library preparation, library quality evaluation, library sequencing, and other procedures. The complete genome sequence of *A. fabrum* L-11 was uploaded to the NCBI database^1^ under BioProject number PRJNA1034247.

### 2.3 Genome assembly and annotation

After obtaining the original data, quality filtering analysis was performed using FastQC v0.11.9 and NanoFilet software [removing low-quality sequences (*Q* < 10) and short sequences (L < 1,000 bp)]. Canu V1.5 software was used to assemble the filtered reads (Koren et al., 2017), Racon V3.4.3 software was employed to correct the assembly results (Vaser et al., 2017), and Circlator V1.5.5 software was used to cycle and adjust the start site (Hunt et al., 2015). Meanwhile, Pilon V1.22^2^ was employed to correct errors with second-generation data for a more accurate genome assembly (Walker et al., 2014). PhiSpy (v2.3) predicted prophages, while CRT (v1.2) was used for CRISPR detection (Bland et al., 2007). Gene prediction was performed using Prodigal software. To obtain the gene function annotation results, the predicted genes were functionally annotated by querying multiple databases, including UniProt, KEGG, GO, RefSeq, TIGERfam, Pfam, Swiss-Prot, COG, and KEGG using BLAST (Fu et al., 2020). Carbohydrate-active enzymes were annotated using the web-based dbCAN2 meta-server (supported by two tools) (Zhang et al., 2018).

### 2.4 Analysis of bacterial pathogenic virulence factor

The virulence factors (VFs) database (VFDB, https://www.mgc.ac.cn/VFs/main.htm) was used to analyze bacterial VFs (Liu et al., 2022). VFDB provides a single platform for storing, searching, retrieving, and updating VF information across different bacterial pathogens (Chen et al., 2005).

### 2.5 Analysis of plasmid and opine types

Genomic information, including tRNA, rRNA, repetitive sequences, GC content, and gene function data, was assembled and analyzed. The Circos V0.66 software was used to create a genomic circle map to examine the positional relationships of genomic components and to obtain precise information about the type of Ti plasmid (Krzywinski et al., 2009).

### 2.6 Genome comparative and OrthoVenn analysis

The genome sequences of *A. radiobacter* standard strain K84 (BioProject: PRJNA13402) and *A. fabrum* standard strains C58 (BioProject: PRJNA283) and 1D132 (BioProject: PRJNA494479) were downloaded from NCBI and compared with the genome sequences, including plasmids, of *A. fabrum* L-11 (BioProject: PRJNA1034247). A total of four strains were analyzed, and genomic information for each strain was obtained using Prodigal software. The OrthoVenn online service was used to identify orthologous gene clusters (Wang et al., 2015), while TBtools (v1.120) was employed to analyze genomic synteny (Chen et al., 2020; Ali et al., 2026). The RAST server was employed for comprehensive automated annotation of the bacterial genomes (Aziz et al., 2008; Islam et al., 2022).

### 2.7 Collinearity analysis and average nucleotide identity calculation

We performed collinearity analysis between the Ti plasmid of L-11 and the corresponding plasmids from strain K84, as well as the pTi1D132 plasmid of strain 1D132. The analysis used TBtools and Mauve software to assess genomic synteny and identify structural variations in gene arrangement, respectively (Darling et al., 2004; Chen et al., 2020). Additionally, we calculated average nucleotide identity (ANI) and digital DNA–DNA hybridization (dDDH) values using JSpecies WS (Richter et al., 2016; Xu et al., 2023). The Genome-to-Genome Distance Calculator (GGDC, v3.0) was employed with default settings for genome comparison (Meier-Kolthoff et al., 2013).

## 3 Results

### 3.1 Whole genome assembly and analysis

**Figure 1:**
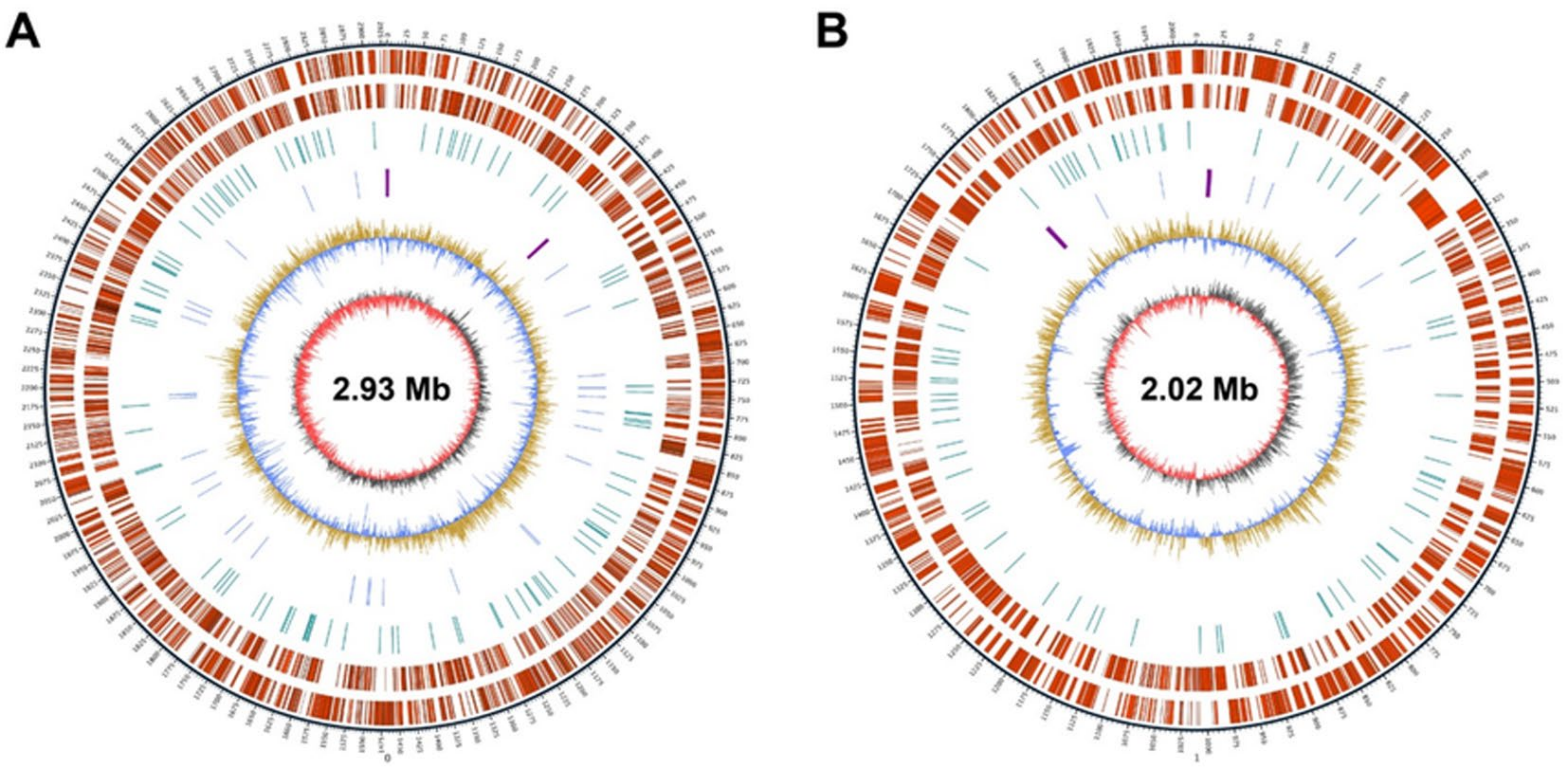
Circular map of the blueberry crown gall pathogen *Agrobacterium fabrum* L-11 chromosomes. The outermost circle shows the size of the chromosome. **(A)** Linear chromosome and **(B)** circular chromosome.

In this study, the entire genome of *A. fabrum* L-11 was sequenced, assembled, and analyzed. A total of 1.07 Gb of raw reads was obtained from genome sequencing of *A. fabrum* L-11 on an Illumina platform through *de novo* assembly (Table 1). The estimated genome size of *A. fabrum* L-11 is approximately 5.70 Mb, comprising a linear chromosome of 2.93 Mb and a circular chromosome of 2.02 Mb (Figure 1). A notable feature of the *A. fabrum* L-11 genome is its collection of four distinct plasmids (see Supplementary Figure S1), which is uncommon for *A. fabrum* strains that usually carry only two or three plasmids. These four plasmids range in size from 0.12 to 0.232 Mb, with a total plasmid genome size of approximately 0.743 Mb, making up more than half of the entire genome. This unusually high number of plasmids indicates significant horizontal gene transfer and a high level of metabolic flexibility. The genome is complete with no gaps, and the GC content of *A. fabrum* L-11 is approximately 59.0%. A total of 5,385 genes were identified, including 5,245 protein-coding sequences (CDS) and 140 RNA genes (54 tRNAs, 12 rRNAs, and 74 other non-coding RNAs). The antisense codons and amino acids of the 54 tRNAs and 74 other RNAs are grouped into 43 families, such as TPP and FMN. Additionally, the genome encodes 471 secreted proteins, 471 signal peptides, and 1,296 transmembrane proteins. No CRISPR structures were identified in the genome of *A. fabrum* L-11.

**Table 1:** Quality statistics of genome sequencing data of blueberry crown gall pathogen *Agrobacterium fabrum* L-11.

Features	Raw reads	Clean reads
Number of reads	136,100	91,278
Total bases (bp)	1,252,940,445	1,074,651,376
N50 length (bp)	15,265	16,062
N90 length (bp)	4,826	5,962
Mean read length (bp)	9,206	11,773
Maximum read length (bp)	100,698	100,698
Mean quality score (*Q*-score)	8.74	9.74

Raw reads represent data directly from the sequencing platform; clean reads represent data after quality filtering (removal of adapters, low-quality bases, and short reads).

### 3.2 Functional annotation

**Figure 2:**
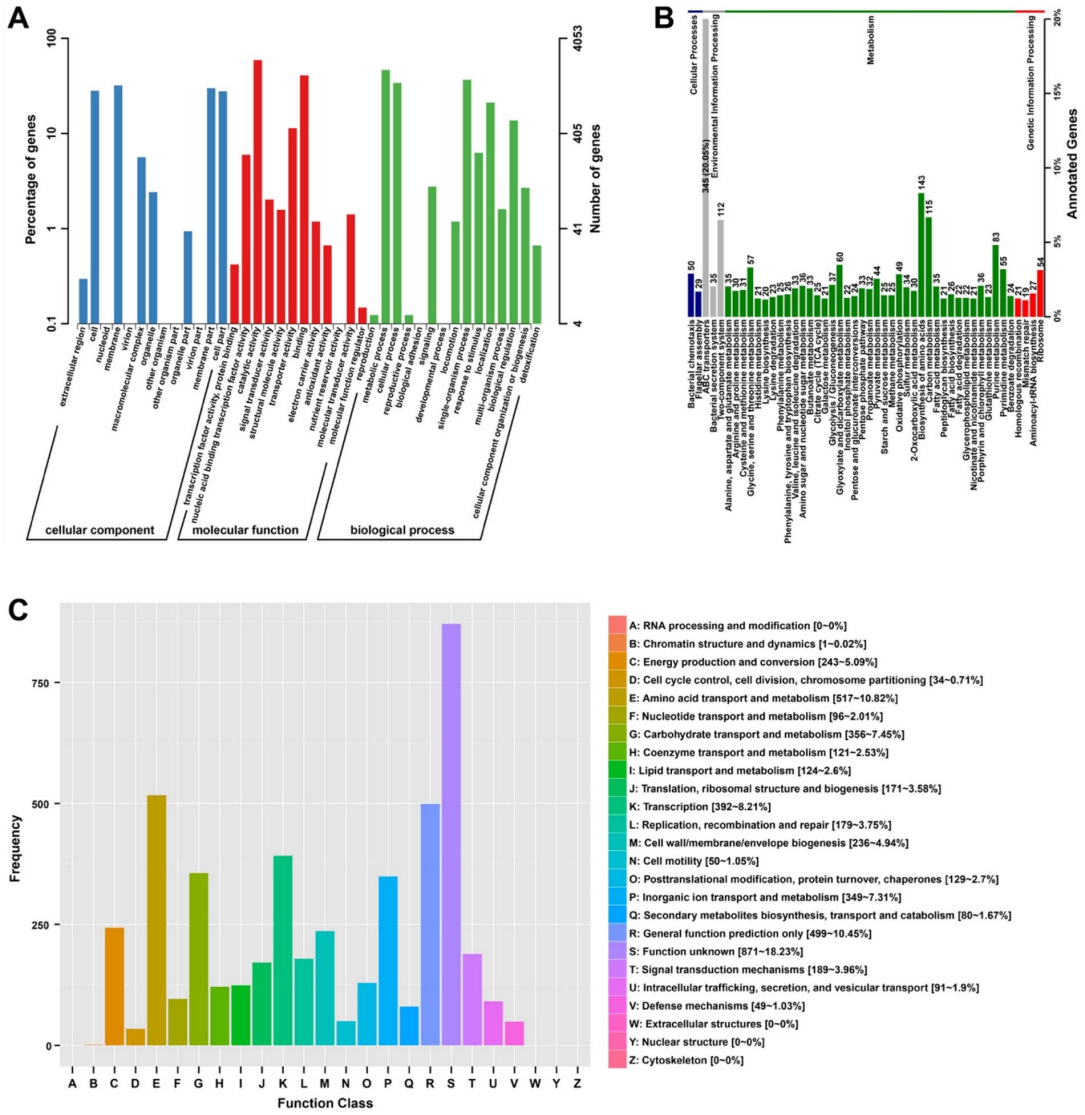
Functional annotation classification of the *Agrobacterium fabrum* L-11 genome. **(A)** Gene ontology (GO) classification showing the distribution of coding sequences across three categories: biological process, molecular function, and cellular component. **(B)** KEGG pathway enrichment highlighting dominant metabolic pathways. **(C)** EggNOG categories illustrating the functional distribution of genes.

A total of 5,245 CDS were predicted in the genome of *A. fabrum* L-11, representing 97.53% of the predicted ORFs. To obtain detailed gene function information for *A. fabrum* L-11, the genome was annotated using seven databases: GO, KEGG, eggNOG, NR, Pfam, SwissProt, and TrEMBL (Table 2). The annotation results were obtained by linking genes with their respective functional information. In the CC category, most genes were annotated as part of the membrane or membrane components. In the MF category, the main annotations involved catalytic activity and binding functions. In the BP category, the majority of annotated genes participated in metabolic processes and single-organism processes (Figure 2A). KEGG functional annotation was conducted to systematically analyze the metabolic pathways and cellular functions of *A. fabrum* L-11 gene products and compounds. The analysis showed that the genome was assigned to 48 metabolic pathways (Figure 2B). The most enriched pathway was ABC transporters, followed by amino acid biosynthesis and carbon metabolism, with 345, 143, and 115 genes, respectively, representing 12.46, 5.16, and 4.15% of all genes. Additionally, 4,686 genes were annotated across 25 eggNOG classification systems (Figure 2C). Among these, 871 were classified as unknown genes, making up the largest group (18.23%), followed by amino acid transport and metabolism with 517 genes, accounting for 10.82% of the annotated genes.

**Table 2:** Summary of functional annotation results for *Agrobacterium fabrum* L-11 genome.

Database	Number of annotated genes
eggNOG Annotation	4,686
GO Annotation	4,052
KEGG Annotation	2,768
NR Annotation	5,215
Pfam Annotation	4,601
Swissprot Annotation	3,181
TrEMBL Annotation	3,181
All Annotated	5,220

The table displays the number of protein-coding genes annotated against seven major databases. eggNOG, evolutionary genealogy of genes; GO, Gene Ontology; KEGG, Kyoto Encyclopedia of Genes and Genomes for metabolic pathways and functional modules; NR, NCBI non-redundant protein database; Pfam, protein families and domains, and SwissProt/TrEMBL, curated and automated protein sequences.

### 3.3 Carbohydrate-active enzymes (CAZymes)

**Figure 3:**
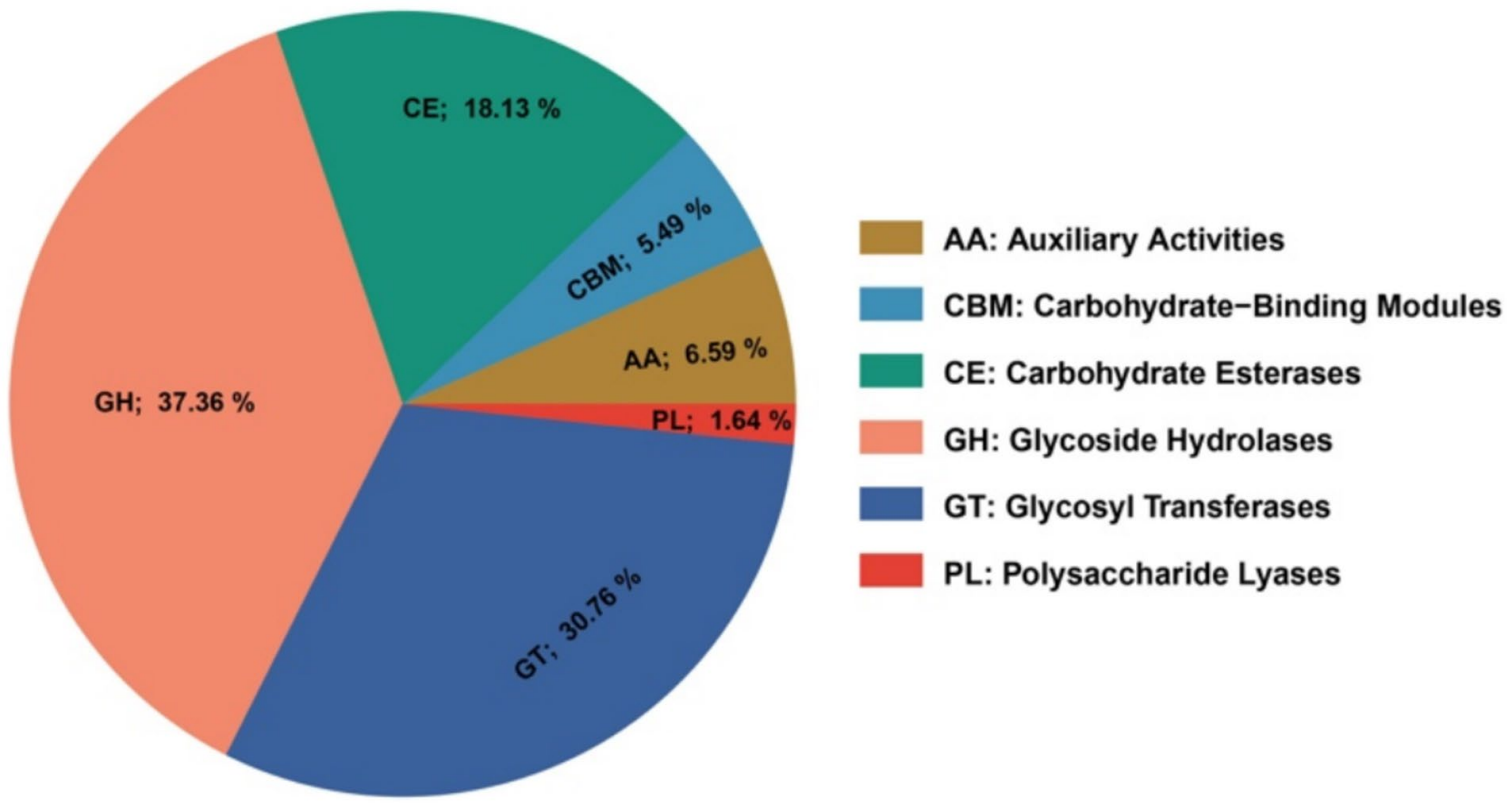
A pie chart illustrates the carbohydrate-active enzyme (CAZyme) encoded in the *Agrobacterium fabrum* L-11 genome.

The genome of *A. fabrum* L-11 encodes 364 carbohydrate-active enzymes (CAZymes) across six main families, including Glycoside Hydrolases (GHs; 37.36%), Glycosyltransferases (GTs; 30.77%), Polysaccharide Lyases (PLs; 1.65%), Sugar Esterases (CEs; 18.13%), Oxidoreductases (AAs; 6.59%), and Carbohydrate Binding Domains (CBDs; 5.50%) (Figure 3). The abundance and diversity of these enzymes offer several benefits for host infection and survival. This range of enzymes supports infection through multiple mechanisms. GHs degrade blueberry cell wall components (cellulose, hemicellulose, and pectin), allowing tissue invasion and nutrient absorption. GTs produce exopolysaccharides essential for bacterial attachment, biofilm formation, and defense against plant defenses. Compared to reference strains, L-11 has a wider array of GH and GT families, which may help explain its host specificity and enhanced ability to colonize blueberry tissues.

### 3.4 Classification of bacterial pathogenic virulence factors

**Figure 4:**
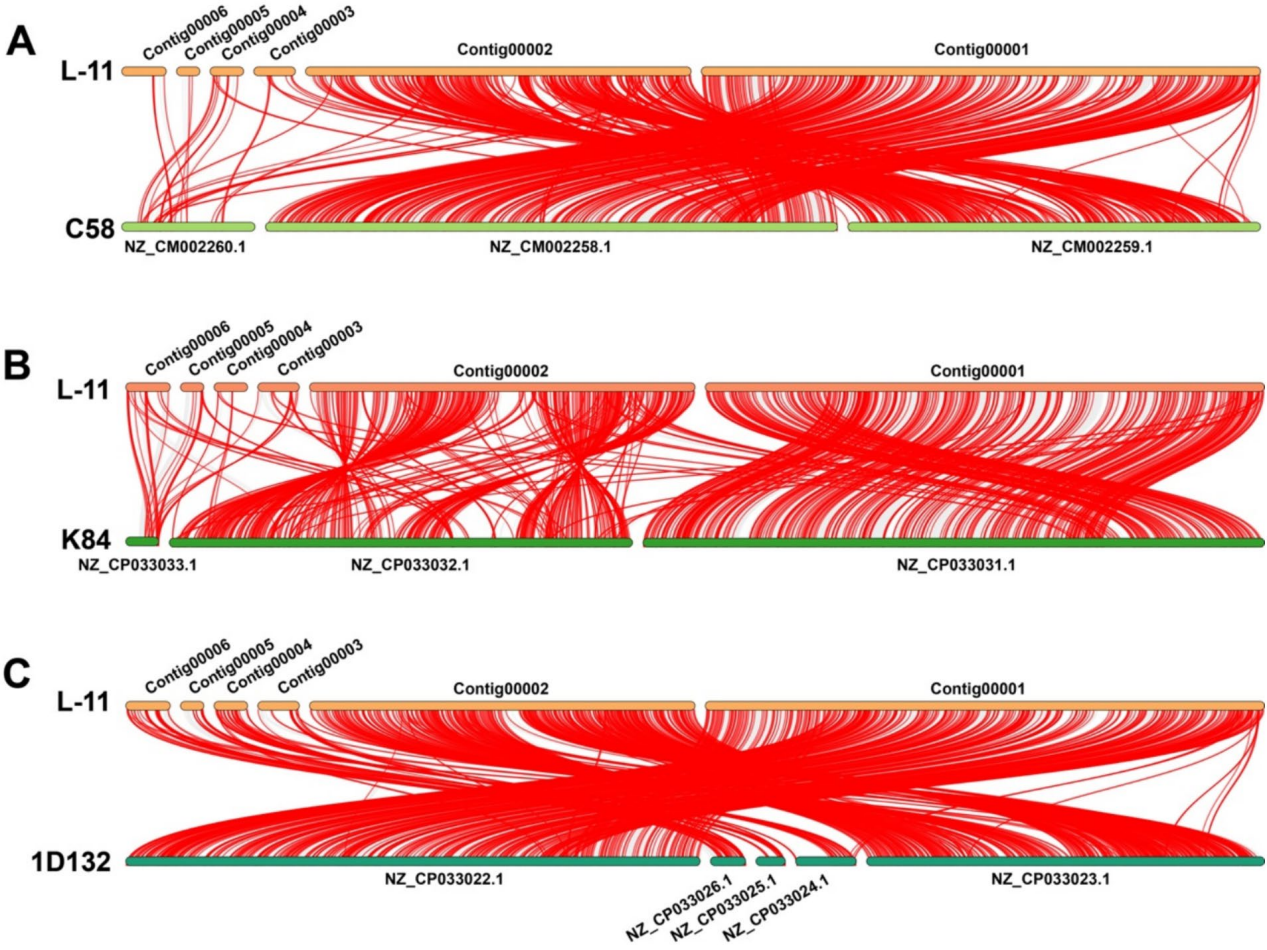
LINNE collinearity analysis of virulence-associated genes in *Agrobacterium fabrum* L-11 compared with reference strains. **(A)** Collinearity analysis between L-11 and C58 based on VFDB genes. **(B)** Collinearity analysis between L-11 and K84 based on VFDB genes. **(C)** Collinearity analysis between L-11 and 1D132 based on VFDB genes. Here, L-11 refers to *A. fabrum* L-11, C58 to *A. fabrum* C58, K84 to *A. radiobacter* K84, and 1D132 to *A. fabrum* 1D132. L-11 consists of Contig00001: a circular chromosome; Contig00002: a linear chromosome; Contig00003: plasmid; Contig00004: plasmid; Contig00005: plasmid; Contig00006: Ti plasmid. C58 includes NZ_CM002259.1: a circular chromosome; NZ_CM002258.1: a linear chromosome; NZ_CM002260.1: Ti plasmid. K84 has NZ_CP033031.1: a chromosome; NZ_CP033032.1: a chromosome; NZ_CP033033.1: a plasmid. 1D132 contains NZ_CP033022.1: a chromosome; NZ_CP033023.1: a chromosome; NZ_CP033024.1: a plasmid; NZ_CP033025.1: a plasmid; NZ_CP033026.1: a plasmid.

We analyzed the VF repertoire in the genome of *A. fabrum* L-11 using VFDB; a total of 1,071 VFs were classified and organized based on their functional differences. The representative names and IDs for each functional VF are listed in Supplementary Table S2. The VFs of *A. fabrum* L-11 were categorized as follows: acriflavine resistance protein, transcriptional regulator, ABC transporter, flagella-related genes, Type III secretion system, Type IV secretion system, and genes associated with lipopolysaccharides. The types of VF genes in *A. fabrum* L-11 matched those of other strains but included many inverted regions and a few deletion regions (Figure 4).

### 3.5 Ti plasmid analysis and comparison of the Ti plasmid map and C58 pathogenic core gene

The tumor-inducing (Ti) plasmid pTiL11 is a circular DNA molecule measuring 231,752 bp (Supplementary Figure S2). The plasmid contains the T-DNA region, Vir region, *ChsC*, *mas1*, *ags*, and *agcA* genes involved in opine synthesis, with the opine being Agropine. Compared to *A. fabrum* C58, which produces Nopaline as its opine, the Ti plasmid core genes include *virB7*, *virD3*, *virE1*, *traA*, and *traM*, while genes such as *tms*, *repC*, *hyuA*, *accD*, and *accE* are missing (Supplementary Table S3). Based on the relative positions of *tra* and *trb* genes and the Vir region in *A. tumefaciens* plasmid, *A. fabrum* L-11 is identified as having a type II plasmid, which includes ChsC (agropine synthesis conjugase), MAS1 (Agropine synthesis reductase), and AGS (agropine) (Supplementary Figure S3).

### 3.6 Collinearity analysis of Ti plasmid

**Figure 5:**
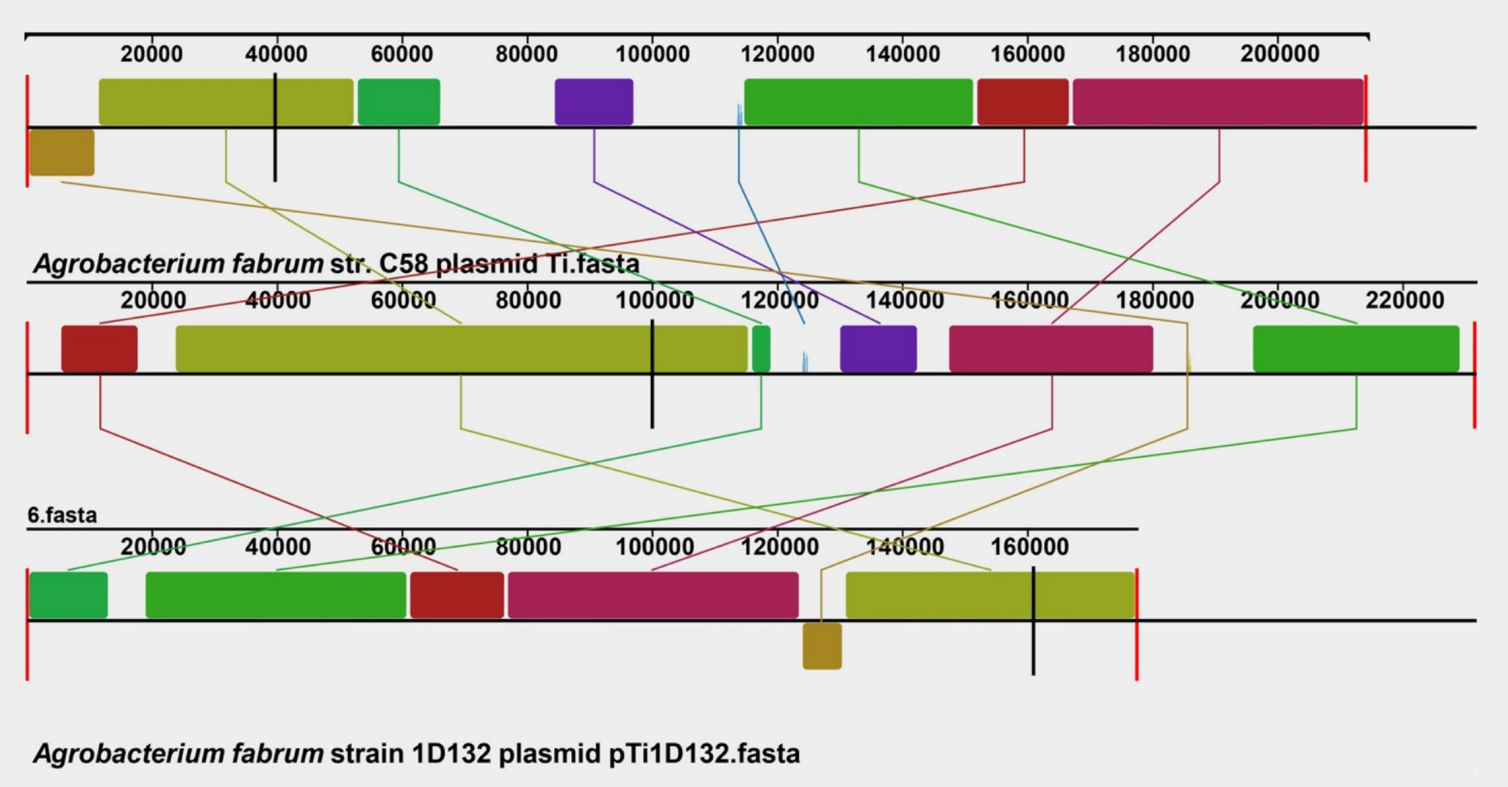
Collinearity analysis of the *Agrobacterium fabrum* L-11 Ti plasmid with reference plasmids from strains K84 and 1D132.

The collinear relationship among the *A. fabrum* L-11 Ti plasmid, *A. radiobacter* K84 plasmid pAt12D1, and *A. fabrum* 1D132 plasmid pTi1D132 is shown in Figure 5. The results indicate that the plasmids of the three strains differ. The plasmid sequence of L-11 is similar to that of the C58 plasmid but contains several inverted regions. Compared to the 1D132 plasmid pTi1D132, the L-11 plasmid has many inverted regions and a few deletion regions.

### 3.7 Comparative genomic analysis

**Figure 6:**
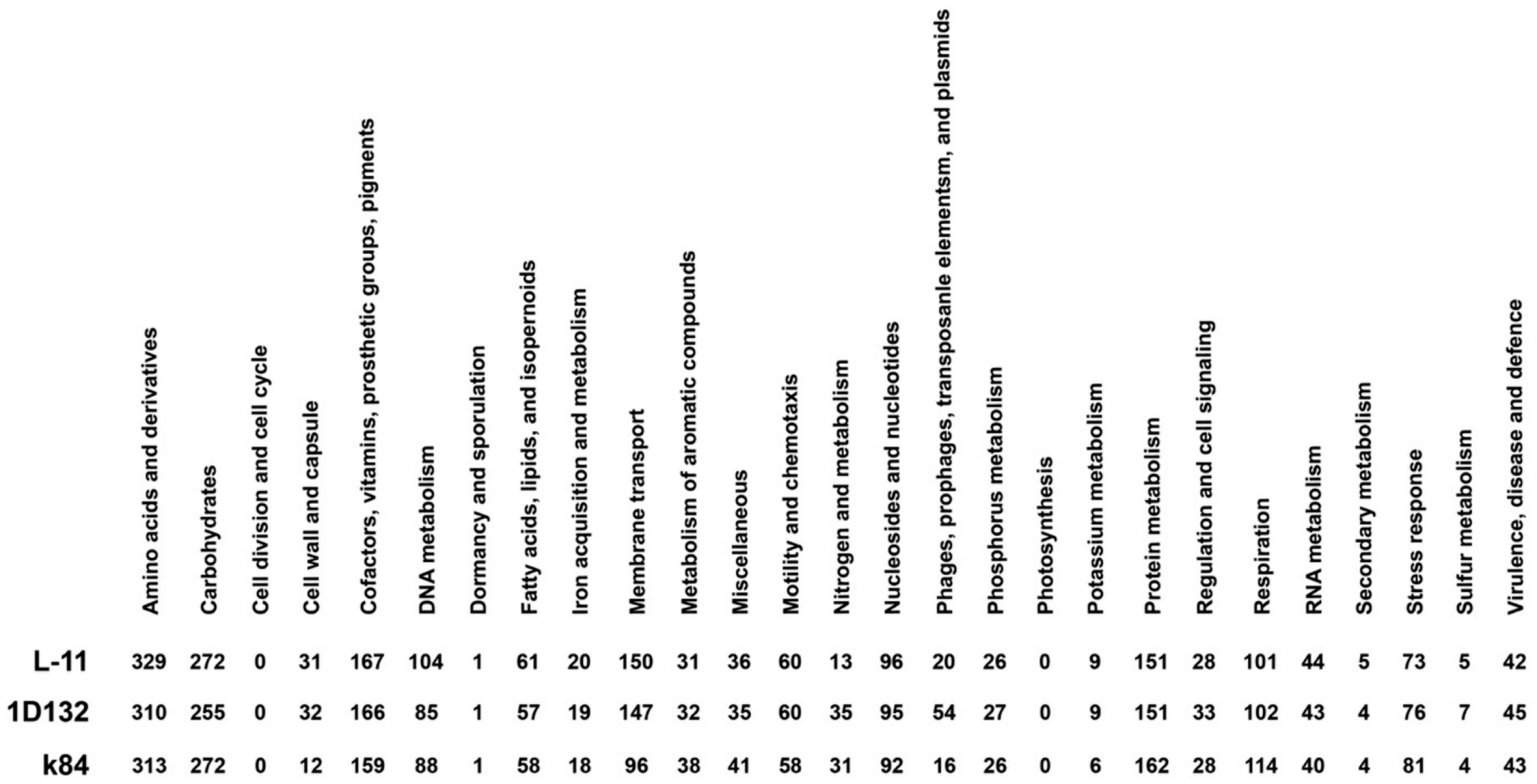
Comparative analysis of subsystem functional categories in *Agrobacterium* strains L-11, 1D132, and K84 using RAST annotation.

The genome of L-11 was compared to *the genomes of Agrobacterium* strains C58, K84, and 1D132 (Supplementary Table S4). Comparative analysis showed that the number of chromosomes and plasmids varied among the four strains, while their GC content remained consistent. Additionally, the number of different RNAs varied. Genomic analysis indicated that L-11 is closely related to strain 1D132 and more distantly related to K84. The RAST and SEED-Viewer databases were used to compare genomes based on this information (Figure 6). The number of subsystems involved in amino acids and derivatives, as well as DNA metabolism, was higher in strain L-11 compared to 1D132 and K84. Conversely, the number of subsystems responsible for nitrogen metabolism in strain L-11 was lower than in 1D132 and K84.

### 3.8 Average nucleotide identity calculation (ANI) and OrthoVenn analysis

**Figure 7:**
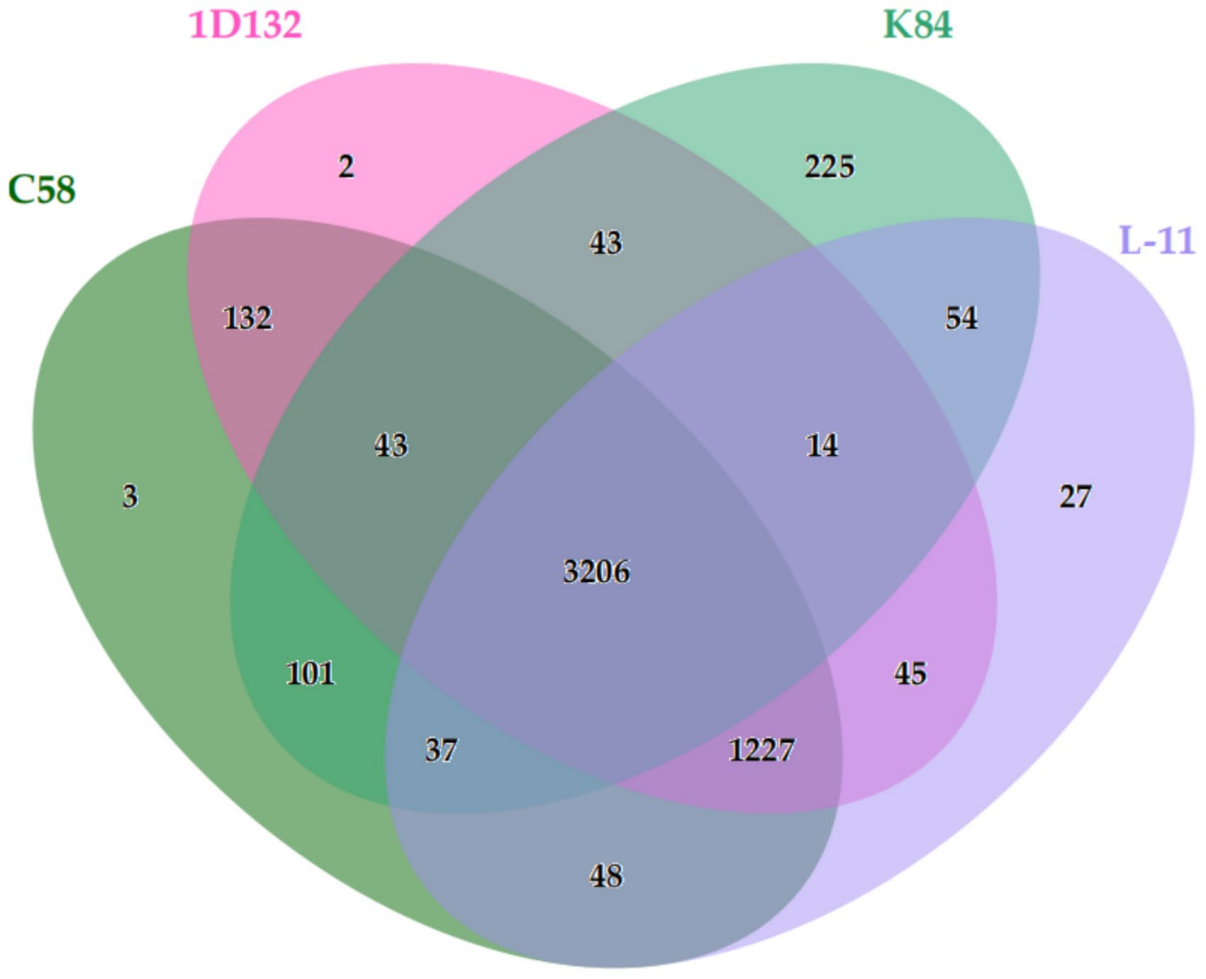
Venn diagram showing the shared orthologous protein clusters among the four *Agrobacterium* strains L-11, C58, 1D132, and K84.

The ANI is mainly used to evaluate DNA similarity across the entire genome between species. We performed dDDH and ANI analyses on strain L-11 and other *Agrobacterium* strains C58, 1D132, and K84 (Table 3). The digital DNA–DNA hybridization (dDDH) values among the strains were calculated using different methods in this study. The dDDH values of strains C58 and K84 relative to L-11 were 57.8 and 61.9%, respectively. Strain 1D132 was closely related to L-11, with a dDDH value of 84.3%, exceeding 70%. Additionally, the ANIb values of strains C58 and K84 relative to L-11 were 85.59 and 87.82%, respectively, which are below the 95% species boundary threshold. The ANIb value of L-11 relative to 1D132 was 96.88%. When the ANIb and dDDH values between strains are above their respective thresholds, meaning ANIb is 95% or higher and dDDH is 70% or higher, it indicates that the strains belong to the same species. The OrthoVenn online tool identified orthologous gene clusters across the three genomes. The results showed that 3,206 gene clusters were shared among the four strains. Meanwhile, 3, 2, 225, and 27 genes were unique to C58, 1D132, K84, and L-11, respectively (Figure 7).

**Table 3:** Average nucleotide identity (ANI) and digital DNA–DNA hybridization (dDDH) values comparing *Agrobacterium fabrum* L-11 to reference strains.

Genome	Query genome of L-11	
	ANI values [aligned nucleotides] (%)	dDDH values (%)
1D132	96.88 (88.91)	84.3
C58	85.59 (75.61)	57.8
K84	87.82 (77.58)	61.9

ANI and dDDH values indicate the pairwise comparisons of given genomic sequences with the genome of strain L-11. Here, first value: mean nucleotide identity across aligned regions and value in brackets: percentage of the genome that aligned.

### 3.9 Collinearity and synteny analysis of the linear chromosome

**Figure 8:**
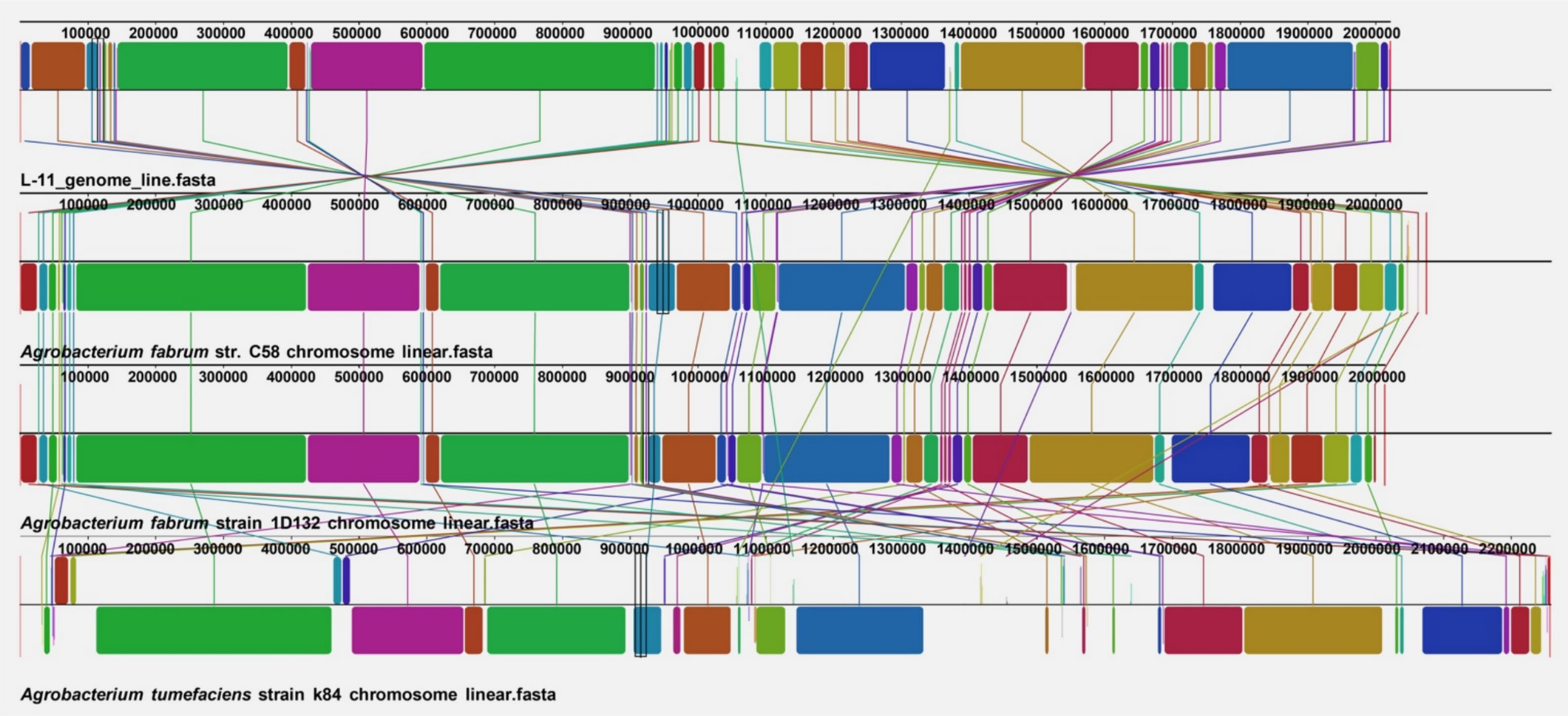
LINNE collinearity analysis of the *Agrobacterium fabrum* L-11 genome compared with reference strains C58, 1D132, and K84.

To examine the evolutionary relationships and genomic rearrangements among *Agrobacterium* strains, we performed a whole-genome alignment of the linear chromosomes from *A. fabrum* L-11 and the reference strains C58, 1D132, and K84 (Figure 8). The linear chromosomes of L-11 and 1D132 showed high collinearity, with 91.2% of the L-11 genome covered by 16 conserved locally collinear blocks (LCBs). Most of these blocks had the same orientation, with only three inversions identified (shown by blocks below the line). The average LCB length was 115.3 kb, indicating strong structural conservation. Alignment with C58 revealed 55 LCBs, covering 71.4% of the L-11 linear chromosome. Several large inversions were observed, especially in the 300–700 kb region, which includes chemotaxis and flagellar genes, and in the 1.2–1.5 Mb region, which contains metabolic operons. The mean LCB length was 26.2 kb, indicating more frequent genetic rearrangements. The linear chromosome of K84 showed limited synteny with L-11, with 156 LCBs covering only 37.8% of L-11’s genome. The average length of these LCBs was just 4.9 kb, pointing to significant fragmentation and rearrangement. This supports the idea that K84 is a different species, *A. radiobacter*, and lacks the linear chromosome structure typical of *A. fabrum.*

## 4 Discussion

Blueberries are native to North America, and their production and consumption are growing worldwide (Banerjee et al., 2020). Blueberries provide not only nutritional and health benefits but also neuroprotective effects against brain aging, cardiovascular advantages by strengthening the heart, anticancer properties, vasoprotective effects by relaxing blood vessels, and immune-boosting benefits in humans (Ma et al., 2018; Wood et al., 2019; Silva et al., 2020). Therefore, blueberries are ranked among the top five healthy foods for humans (Silva et al., 2020). Blueberry farming has become a global industry to meet the increasing demand. As a result, the globalization of the blueberry industry has facilitated the spread of diseases and the introduction of foreign pests into both existing and new blueberry-growing regions (Drummond et al., 2008). Crown gall disease, caused by *Agrobacterium fabrum*, poses a major threat to worldwide blueberry production (Kuzmanović et al., 2019). In this study, we performed comparative genomic analysis and whole-genome sequencing of *A. fabrum* L-11, which offered new insights into its evolutionary divergence from other *Agrobacterium* strains, its virulence factors, and its genomic structure.

The complete genome sequencing of *A. fabrum* L-11 has revealed a unique genomic structure that distinguishes this blueberry isolate from other previously studied *Agrobacterium* strains. *A. fabrum* L-11’s genome is 5.70 Mb in size with a GC content of 59.0%, which is similar to other *Agrobacterium* species (Slater et al., 2009). The identification of four large plasmids, ranging from 0.12 to 0.232 Mb, with a total plasmid content of 0.743 Mb, is a notable feature. This more extensive plasmid complement significantly exceeds the 2–3 plasmids usually observed in other *A. fabrum* strains like C58 (which has 2 plasmids totaling 0.43 Mb) and 1D132 (which has 3 plasmids totaling 0.56 Mb) (Goodner et al., 2001; Ormeno-Orrillo et al., 2015).

The presence of four large plasmids, collectively larger than the chromosomes, suggests that *A. fabrum* L-11 has experienced significant horizontal gene transfer and may have gained enhanced metabolic capabilities. The multipartite genome structure, consisting of a linear chromosome of 2.93 Mb, a circular chromosome of 2.02 Mb, and four plasmids, including a 0.232 Mb Ti plasmid, is consistent with the distinctive genomic architecture of the Rhizobiaceae family (Slater et al., 2009). In the Rhizobiaceae (Ormeno-Orrillo et al., 2015), this multipartite organization is believed to support host adaptation and ecological diversity; this genomic structure is linked to the unique variation observed in previous studies (Du et al., 2023). The virulence and environmental adaptability of the strain likely result from the 5,385 genes and 5,245 coding sequences (CDS) predicted by functional annotation, indicating a genetic potential for various metabolic and pathogenic activities (Hwang et al., 2013; Vinnik et al., 2020). Our observation that the linear chromosome experiences more rearrangements than the circular chromosome supports this theory. It suggests that the linear chromosome could serve as a basis for genomic innovation, while the circular chromosome preserves essential housekeeping functions through stronger purifying selection.

The combined use of ANI analysis, dDDH calculations, OrthoVenn clustering, and synteny analysis offers a comprehensive understanding of the evolutionary relationships between *A. fabrum* L-11 and reference strains (Qiu et al., 2023). We identified both shared traits and unique genomic differences in *A. fabrum* L-11 compared to reference strains K84, C58, and 1D132 (Slater et al., 2009; Du et al., 2023). Significant differences were observed in chromosomal number, plasmid patterns, and RNA gene richness, even though genome sizes and GC content were similar, indicating a shared evolutionary origin. These strain-specific genetic traits likely reflect adaptive processes that influence host-pathogen interactions and ecological niche specialization (Ventura et al., 2007). The genome includes a circular chromosome, a linear chromosome, and four plasmids, all containing a Ti plasmid, which makes it relatively complex. *A. fabrum* L-11 differs from K84, C58, and 1D132 in chromosome and plasmid counts, RNA levels, virulence factors, and some core genes compared to other *Agrobacterium* strains. According to Dos Santos et al. (2023), current taxonomic standards for classifying bacterial species are based on genomic similarity thresholds, with the main criteria being Average Nucleotide Identity (ANI) > 95% and digital DNA–DNA hybridization (dDDH) > 70%. Therefore, bacterial species are classified at the genomic level using ANI and DDH as benchmarks (Qiu et al., 2023). Our research shows that *A. fabrum* L-11 and *A. fabrum* 1D132 have 96.88% ANI and 84.3% dDDH similarity, respectively, supporting their classification as the same species. Conversely, reference strains C58 (85.59%) and K84 (87.82%) exhibited notably lower ANI values, indicating that although they belong to the same phylogenetic group within the Agrobacterium genus, they are different species. These genomic comparisons strongly support placing strain L-11 within the *A. fabrum* species complex.

Analysis utilizing the Virulence Factors Database (VFDB) identified 1,071 potential virulence-related genes in *A. fabrum* L-11. These include genes for secretion systems, ABC transporters, flagellar assembly, and lipopolysaccharide biosynthesis clusters. The presence of complete Type III, Type IV, and Type VI secretion systems indicates advanced machinery for host interaction and interbacterial competition (Alfano and Collmer, 2004; Czolkoss et al., 2021). The genome of *A. fabrum* L-11 includes essential virulence factor genes, such as exsA for type III secretion, virD4, virB1, virB4, and virB9 for type VI secretion, the hitC iron (III) transporter genes of the ABC family, and genes involved in lipopolysaccharide synthesis and anti-acriflavine protein production. This indicates that strain L-11 has all the necessary components for efficient T-DNA processing and transfer (Christie, 2004). The presence of these core virulence genes, even after genomic rearrangements, indicates strong purifying selection on key pathogenicity factors. The Type III secretion system (T3SS), similar to those found in plant pathogens such as *Pseudomonas syringae* and *Xanthomonas campestris*, can deliver effector proteins that suppress plant immune responses (Alfano and Collmer, 2004). The presence of T3SS genes in L-11 indicates that, like other *Agrobacterium* strains, it uses various strategies to evade host defenses. Likewise, the Type VI secretion system (T6SS) is involved in interbacterial competition and may help establish its niche by eliminating competing microbes in the rhizosphere (Gonzalez Moreno and Nishiguchi, 2025).

Functional annotations from multiple databases (GO, KEGG, eggNOG, NR, Pfam, SwissProt, and TrEMBL) offered a comprehensive view of the metabolic and functional capabilities of *A. fabrum* L-11. Our analysis showed numerous genes related to molecule binding (22.4%), membrane processes (32.1%), and catalytic activities (28.7%), indicating a strong cellular system for host contact and environmental sensing (Yang and Hinner, 2015; Zhukov and Popov, 2023). KEGG pathway analysis revealed that ABC transporters are the most significantly enriched metabolic pathway, involving 345 genes (12.46% of annotated genes), followed by amino acid biosynthesis with 143 genes (5.16%) and carbon metabolism with 115 genes (4.15%). The remarkably large ABC transporter repertoire suggests enhanced capabilities for nutrient uptake and xenobiotic export, likely improving ecological adaptability and host colonization efficiency (Liu et al., 2020). These transporters may help acquire various nutrients from the blueberry rhizosphere and export plant-derived antimicrobial compounds, giving them a competitive edge during infection (Tanaka et al., 2018). The eggNOG classification showed that 871 genes (18.23%) were assigned to unknown functions, suggesting that a significant portion of the genome could encode new strain-specific adaptations. Amino acid transport and metabolism (517 genes, 10.82%) was the largest functional category identified, emphasizing the importance of nitrogen metabolism in this pathogen’s plant-associated lifestyle.

The genome of *A. fabrum* L-11 contains 364 carbohydrate-active enzymes (CAZymes), emphasizing its complex carbohydrate metabolism. Consistent with findings from similar strains, our study identified all major CAZyme categories, including glycoside hydrolases (GHs; 37.36%), glycosyl transferases (GTs; 30.77%), and carbohydrate esterases (CEs). This CAZyme repertoire is much larger than what is usually found in Agrobacterium strains and approaches the complexity seen in specialized plant pathogens like *Xanthomonas* and *Ralstonia* (Tanaka et al., 2018). According to Bateman and Basham (1976), this enzyme profile provides two main functional advantages: (1) increased ability to break down plant cell walls, facilitating invasion, colonization of host tissue, and tumor development; and (2) metabolic versatility for using various carbohydrate sources (Bateman and Basham, 1976). After tumors develop, bacteria grow in intercellular spaces and can access glucose, xylose, and other sugars released from plant cell walls. The diverse substrate range of CAZymes in L-11 shows this strain’s capacity to utilize various plant-derived carbohydrates, giving it an advantage in both the rhizosphere and tumor environments (Gavande et al., 2023). The main factor influencing virulence in pathogenic strains of *Agrobacterium* is the tumor-inducing (Ti) plasmid (Christie, 2004). The Ti plasmid (231,752 bp, 56.2% GC) has the typical mosaic structure of Ti plasmids, including separate functional modules for replication, opine breakdown, T-DNA, virulence, and conjugative transfer. Its lower GC content compared to the chromosomes (59.0%) suggests a likely foreign origin through horizontal gene transfer, aligning with the evolutionary history of Ti plasmids (Hooykaas, 2023). The detection of the Ti plasmid in L-11 indicates that this plasmid can transfer, potentially helping spread virulence factors to other bacteria within the tumor environment. Additionally, the presence of several toxin-antitoxin systems helps maintain the plasmid by eliminating plasmid-free daughter cells after division, thereby ensuring these essential virulence genes are preserved even without ongoing selective pressure (Zatyka and Thomas, 1998).

## 5 Conclusion

In conclusion, genome sequencing and comparative analysis have clarified the genetic structure, pathogenicity mechanisms, and ecological adaptations of *Agrobacterium fabrum* L-11. The presence of unique virulence factors and Ti plasmid genes may explain why *A. fabrum* L-11 can infect blueberry plants. The genomic analysis showing that *A. fabrum* L-11 has diverse metabolic abilities indicates it is well-prepared to succeed in different environmental niches. *Genomic differences between A. fabrum* L-11 and other well-studied Agrobacterium strains highlight the ongoing evolution within the genus. These findings lay a solid groundwork for further research into the pathogenicity of bacteria responsible for blueberry crown gall. They could also help develop more effective disease management strategies for blueberry production. Future studies should focus on comparing transcriptomic profiles during infection, validating key virulence factors, and exploring potential biocontrol methods tailored to *A. fabrum* L-11’s unique features.

## Data Availability

The datasets presented in this study can be found in online repositories in the NCBI public database under BioProject: PRJNA1034247.

## Footnotes

1
https://www.ncbi.nlm.nih.gov/

2
https://github.com/broadinstitute/pilon
