# Supplementary material for: Whole-genome sequencing and comparative genomic analysis of Agrobacterium fabrum L-11 causing crown gall disease in blueberry
Source: Front Microbiol. 2026 May 6;17:1776711. doi: 10.3389/fmicb.2026.1776711 (PMC13191632; doi:10.3389/fmicb.2026.1776711)
Supplement: Supplementary file 1 [file Table_1.DOCX]

| Table S1. Basic information of blueberry crown gall pathogen L-11 genome. | |
| --- | --- |
| Feature | L-11 |
| genome size (bp) | 5,698,038 |
| Contig Number | 6 |
| Gaps Number | 0 |
| GC content (%) | 59.0 |
| Gene | 5385 |
| CDS | 5245 |
| tRNA genes | 54 |
| rRNA genes | 12 |
| other ncRNA | 74 |
| CRISPR | 0 |

| Table S2. The classification of bacterial pathogenicity virulence factor (VFDB). | | |
| --- | --- | --- |
| Classification of VFDB | VFDB gene name | VFDB gene ID |
| Acriflavine resistance protein | *acrA*、*acrB* | GE000004  GE000005 |
| Transcriptional regulator | *ptxR*、*pchR* etc. | GE000012  GE000513 |
| ABC transporter | *hitC iron*(III) etc. | GE000270 |
| Flagellate related genes | *flgD*、*flgB* etc. | GE000799  GE000775 |
| Type III secretion system | *exsA* etc. | GE002319 |
| Type IV secretion system | *virD4*  *virB1*  *virB4*  *virB9* etc. | GE004716  GE004699  GE004702  GE004707 |
| Genes associated with lipopolysaccharides | *lpsB/lpcC* etc. | GE001436  GE003153  GE002473 |

| Table S3. Comparison of Ti plasmid core genes. | | | |
| --- | --- | --- | --- |
| **L-11** | | **C58** | |
| **Gene name** | **Function** | **Gene name** | **Function** |
| *accR* | plasmid conjugation | *accR* | plasmid conjugation |
| *repB* | plasmid replication | *repB* | plasmid replication |
| *traB* | plasmid conjugation | *traB* | plasmid conjugation |
| *traC* | plasmid conjugation | *traC* | plasmid conjugation |
| *traD* | plasmid conjugation | *traD* | plasmid conjugation |
| *traF* | plasmid conjugation | *traF* | plasmid conjugation |
| *traG* | plasmid conjugation | *traG* | plasmid conjugation |
| *traR* | plasmid conjugation | *traR* | plasmid conjugation |
| *trbB* | plasmid conjugation | *trbB* | plasmid conjugation |
| *trbC* | plasmid conjugation | *trbC* | plasmid conjugation |
| *trbD* | plasmid conjugation | *trbD* | plasmid conjugation |
| *trbE* | plasmid conjugation | *trbE* | plasmid conjugation |
| *trbG* | plasmid conjugation | *trbG* | plasmid conjugation |
| *trbH* | plasmid conjugation | *trbH* | plasmid conjugation |
| *trbI* | plasmid conjugation | *trbI* | plasmid conjugation |
| *virB8* | T-DNA transfer | *virB8* | T-DNA transfer |
| *virB9* | T-DNA transfer | *virB9* | T-DNA transfer |
| *virB10* | T-DNA transfer | *virB10* | T-DNA transfer |
| *agcA* | Agropine synthesis cyclase | *accD* | agrocinopine catabolism |
| *ags* | Agropine synthesis cyclase | *accE* | agrocinopine catabolism |
| *ChsC* | Agropine synthesis conjugase | *hyuA* | hydantoin catabolism |
| *mas1* | Agropine synthesis cyclase | *repC* | plasmid replication |
| *traA* | plasmid conjugation | *tms1* | oncogene (auxin biosynthesis) |
| *traG* | plasmid conjugation | *tms2*** | oncogene (auxin biosynthesis Ti only) |
| *traM* | plasmid conjugation | *traI* | plasmid conjugation |
| *trbF* | plasmid conjugation | *traH* | plasmid conjugation |
| *trbJ* | plasmid conjugation | *virA* | T-DNA transfer |
| *trbL* | plasmid conjugation | *virB1* | T-DNA transfer |
| *virB7* | T-DNA transfer | *virB2* | T-DNA transfer |
| *virC1* | T-DNA transfer | *virB4* | T-DNA transfer |
| *virD1* | T-DNA transfer | *virB5* | T-DNA transfer |
| *virD2* | T-DNA transfer | *virB6* | T-DNA transfer |
| *virD3* | T-DNA transfer | *virB11* | T-DNA transfer |
| *virD4* | T-DNA transfer |  |  |
| *virE1* | T-DNA transfer |  |  |

| Table S4. Basic information of blueberry crown gall Pathogens L-11 genome and others *Agrobacterium* genome. | | | | | | | |
| --- | --- | --- | --- | --- | --- | --- | --- |
| **Feature** | genome size (bp) | Chromosomes and plasmids number | GC content (%) | tRNA | rRNA | Other RNA | CRISPR |
| **L-11** | 5,698,038 | 6 | 59.0 | 54 | 12 | 74 | 0 |
| **C58** | 5,674,260 | 3 | 59.0 | 56 | 4 | 26 | - |
| **K84** | 5,636,830 | 3 | 59.5 | 51 | 3 | 23 | - |
| **1D132** | 5,553,114 | 5 | 59.0 | 49 | 4 | 4 | - |


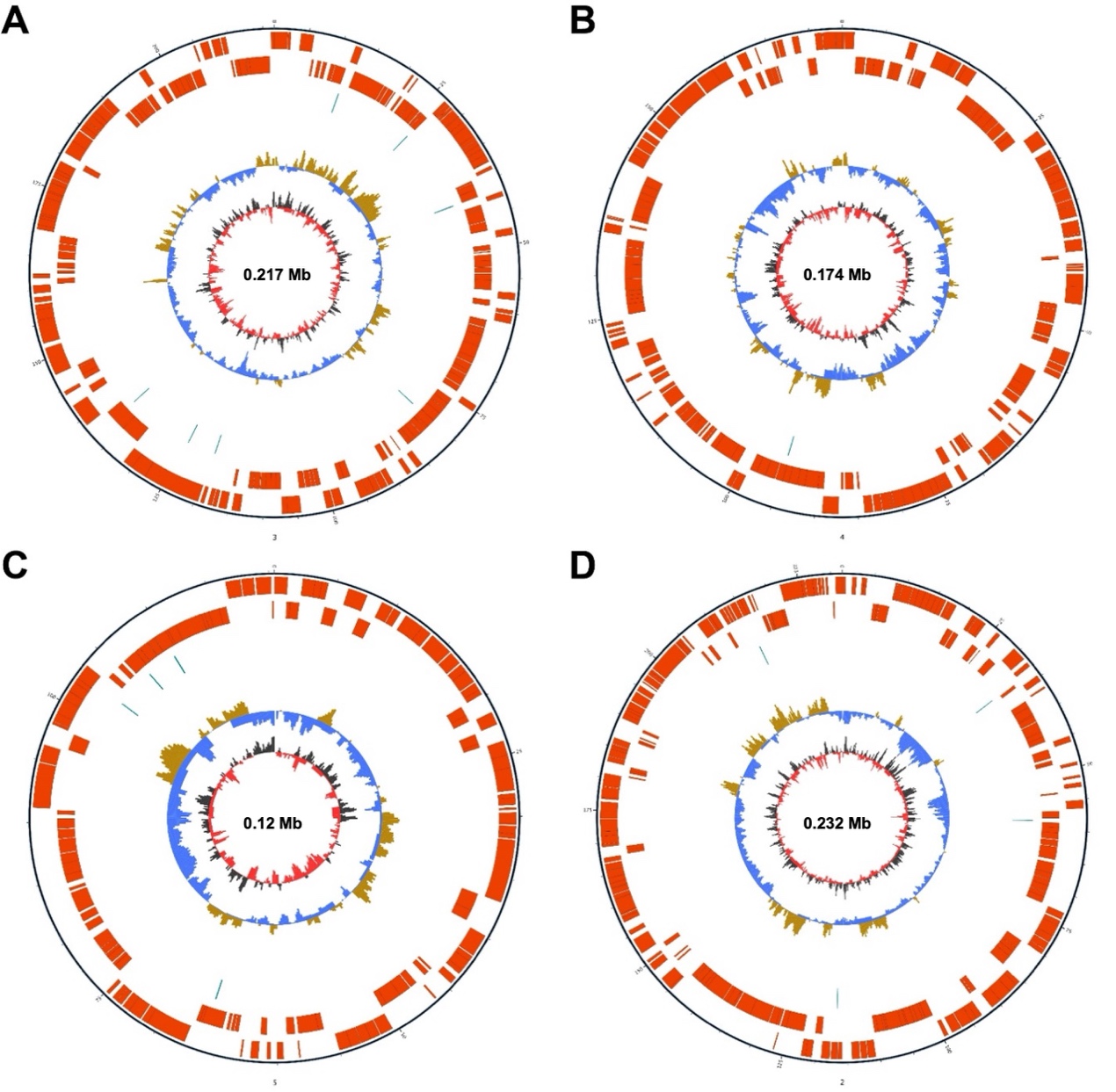


Figure S1: The map of four plasmids in the blueberry crown gall pathogen L-11.


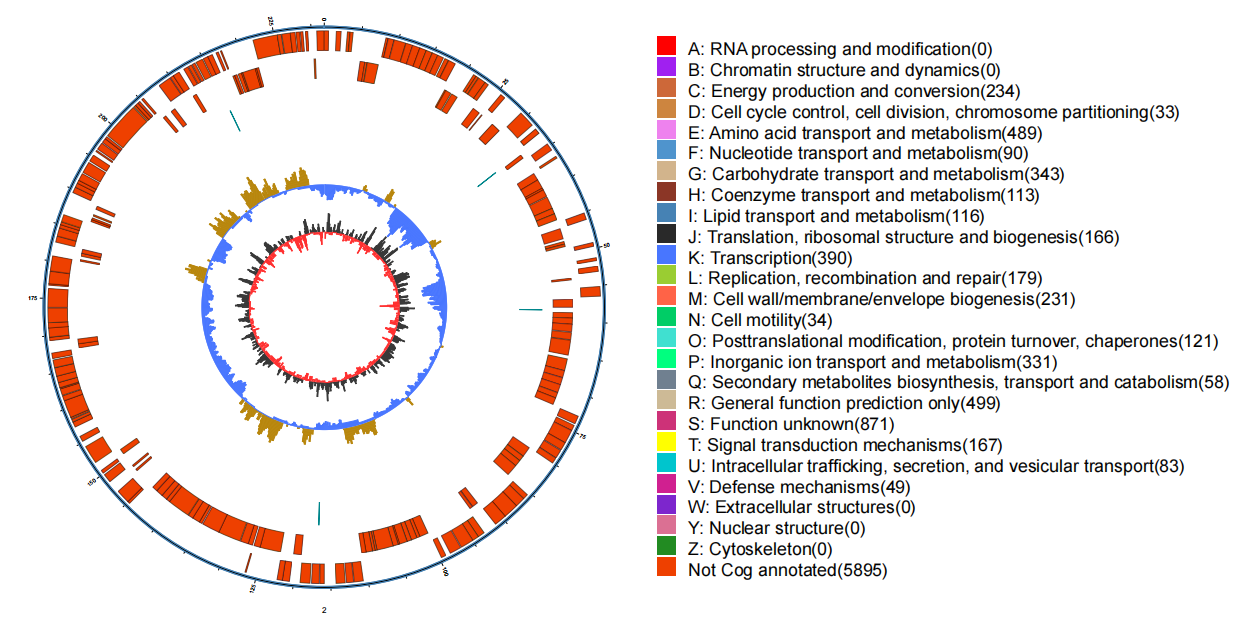


Figure S2. Ti plasmid map of *A. fabrum* L-11.


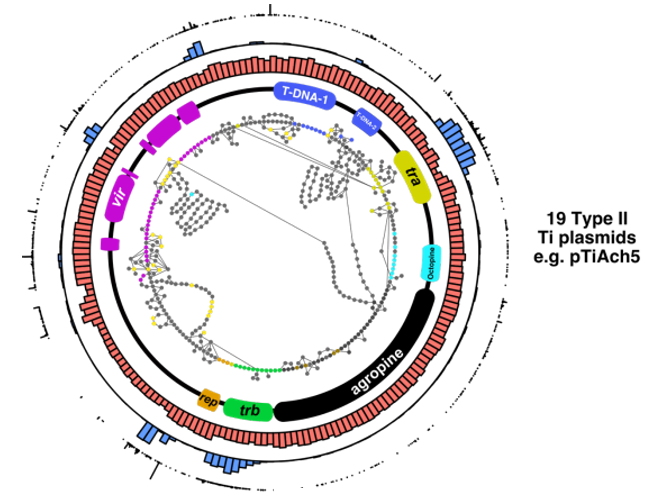


Figure S3. *Agrobacterium fabrum* Type II Ti plasmid.
